# Longitudinal performance of polycaprolactone-based scaffold plus recombinant human morphogenetic protein-2 (rhBMP-2) in large preclinical animal model: 6- versus 12 months

**DOI:** 10.1186/1748-7161-10-S1-O58

**Published:** 2015-01-19

**Authors:** Mostyn RNO Yong, Flavia M Savi, Maria A Woodruff, Siamak Saifzadeh, Geoffrey N Askin, Robert D Labrom, Dietmar W Hutmacher, Clayton J Adam

**Affiliations:** 1 QUT/Mater Paediatric Spine Research Group, Queensland University of Technology, Mater Misericordiae Health Services, Brisbane, Queensland, Australia; 2 Institute of Health and Biomedical Innovation, Queensland University of Technology, Brisbane, Queensland, Australia

## Objectives

There is strong current interest in the use of biodegradable scaffolds in combination with bone growth factors as a valuable alternative to the current gold standard autograft in spinal fusion surgery (Yong et al. 2013). Here we report on 6- vs 12- month data set evaluating the longitudinal performance of a CaP coated polycaprolactone (PCL) scaffold loaded with recombinant human bone morphogenetic protein-2 (rhBMP-2) as a bone graft substitute within a preclinical ovine thoracic spine.

## Material and methods

Twelve sheep underwent a 3-level (T6/7, T8/9 and T10/11) discectomy with randomly allocated implantation of a different graft substitute at each of the three levels; (i) calcium phosphate (CaP) coated polycaprolactone based scaffold plus 0.54mcg rhBMP-2, (ii) CaP coated PCL- based scaffold alone or (iii) autograft (mulched rib head). Fusion assessments were performed via high resolution clinical computed tomography and histological evaluation were undertaken at six (n=6) and twelve (n=6) months post-surgery using the Sucato grading system (Sucato et al. 2004).

## Results

The computed tomography fusion grades of the 6- and 12- months in the rhBMP-2 plus PCL- based scaffold group were 1.9 and 2.1 respectively, in the autograft group 1.9 and 1.3 respectively, and in the scaffold alone group 0.9 and 1.17 respectively. There were no statistically significant differences in the fusion scores between 6- and 12- month for the rhBMP plus PCL- based scaffold or PCL - based scaffold alone group however there was a significant reduction in scores in the autograft group. These scores were seen to correlate with histological evaluations of the respective groups.

## Conclusions

The results of this study demonstrate the efficacy of scaffold-based delivery of rhBMP-2 in promoting higher fusion grades at 6- and 12- months in comparison to the scaffold alone or autograft group within the same time frame. Fusion grades achieved at six months using PCL+rhBMP-2 are not significantly increased at twelve months post surgery.
